# Wenn AIHA nicht alles erklärt: Parvovirus-B19-Infektion und Panzytopenie als differenzialdiagnostische Herausforderung

**DOI:** 10.1007/s00108-025-02018-9

**Published:** 2025-11-25

**Authors:** W. Harms, R. Akdas, C. Jacob, J. Schanz, G. Wulf, N. Brökers

**Affiliations:** 1 Klinik für Hämatologie und medizinische Onkologie, Universitätsmedizin Göttingen, Robert-Koch-Str. 40, 37075 Göttingen, Deutschland; 2 Klinik für Kardiologie und Pneumologie, Universitätsmedizin Göttingen, Göttingen, Deutschland; 3 Institut für Klinische Chemie, Universitätsmedizin Göttingen, Göttingen, Deutschland

**Keywords:** Autoimmunhämolytische Anämie, Hämolyse, Erythropoese, Panzytopenie, Coombs-Test, Autoimmune hemolytic anemia, Hemolysis, Erythropoiesis, Pancytopenia, Coombs test

## Abstract

Eine 48-jährige Patientin mit bekannter autoimmunhämolytischer Anämie (AIHA) stellte sich mit ausgeprägter Anämie und Panzytopenie vor. Trotz typischer Hämolyseparameter und positivem Coombs-Test mit Nachweis von Wärmeantikörpern zeigte sich eine hyporegenerative Erythropoese, sodass in der Zusammenschau von einem kombinierten Geschehen aus gesteigertem Erythrozytenabbau und eingeschränkter hämatopoetischer Kapazität auszugehen war. Die weiterführende Diagnostik ergab den Nachweis einer aktiven Parvovirus-B19-Infektion. Die Fallkonstellation verdeutlicht, dass die Koinzidenz einer AIHA mit einer Parvovirus-B19-Infektion ein atypisches hämatologisches Bild erzeugen und die Diagnosestellung erheblich erschweren kann.

## Abstract (en)

A 48-year-old female patient with known autoimmune hemolytic anemia (AIHA) presented with severe anemia and pancytopenia. Despite typical hemolysis parameters and a positive direct antiglobulin test confirming the presence of warm autoantibodies, erythropoiesis was found to be hyporegenerative. This constellation suggested a combined mechanism of increased erythrocyte destruction and impaired hematopoietic capacity. Further diagnostic workup revealed active parvovirus B19 infection. This case highlights that the coincidence of AIHA and parvovirus B19 infection can lead to an atypical hematologic presentation and significantly complicate the diagnostic process.

## Anamnese

Wir übernahmen eine 48-jährige Patientin mit vorbekannter autoimmunhämolytischer Anämie (AIHA) vom Wärmeantikörpertyp aus einem externen Krankenhaus, in dem sie sich aufgrund zunehmender Schwäche, Rückenschmerzen und Belastungsdyspnoe vorgestellt hatte. Zudem bestand eine unspezifische Schwellung des linken Knies. In der externen Labordiagnostik zeigten sich anfangs nur milde Blutbildveränderungen. Im Verlauf von nur zwei Tagen kam es jedoch zu einer deutlichen Panzytopenie mit zunehmender Anämie und Thrombozytopenie sowie einer Leukopenie bis hin zur Agranulozytose. Sonographisch zeigte sich eine Hepatosplenomegalie. Nebenbefundlich ergab sich nach einmaliger Fieberepisode der Nachweis eines Harnwegsinfekts, welcher antibiotisch mit Ceftriaxon behandelt wurde.

Acht Monate zuvor war erstmals die autoimmunhämolytische Anämie idiopathischer Genese diagnostiziert worden. Im Rahmen der Erstdiagnose war eine Knochenmarkpunktion erfolgt. Hier hatte sich kein Anhalt für einen malignen Befund ergeben. Bei zuvor kompensierter hämolytischer Anämie war bisher keine Therapieindikation gestellt worden. Nun wurde erstmals eine Glukokortikoidstoßtherapie initiiert. Hierauf war kein Ansprechen zu beobachten; die Anämie zeigte sich in den Tagen vor der Übernahme in unsere Klinik rasch progredient.

## Diagnostik

### Klinischer Befund

Bei Übernahme präsentierte sich die Patientin in einem reduzierten Allgemeinzustand. Neben allgemeiner Schwäche und Müdigkeit berichtete sie über eine im Verlauf deutlich zunehmende Belastungsdyspnoe sowie Schwindel bei Mobilisierung. Die zuvor bestehenden harnwegsinfektassoziierten Symptome der Dysurie und Pollakisurie waren nach extern abgeschlossener antibiotischer Therapie regredient. Im körperlichen Untersuchungsbefund fielen eine Blässe sowie eine Ruhetachykardie bis 120 bpm auf.

### Labordiagnostik

Der Hämoglobinwert betrug bei Übernahme der Patientin 5,3 g/dl und fiel im kurzen Intervall bis auf 3,5 g/dl. Als Anzeichen einer intravasalen Hämolyse lagen eine Hyperbilirubinämie, eine Erhöhung der LDH, ein erhöhtes freies Hämoglobin und ein erniedrigtes Haptoglobin vor. MCV und MCH waren normwertig. Ein Substratmangel lag nicht vor (Folsäure, Holotranscobalamin und Eisenstatus unauffällig). Weiterhin zeigte sich die bereits extern auffällige Leukopenie mit Granulozytopenie (formal Aplasie) und Thrombozytopenie, sodass insgesamt eine Panzytopenie vorlag (Tab. 1).

**Tab. 1:** Laborwerte bei Übernahme sowie bei Entlassung der Patientin.

	Referenz	Einheit	Bei Übernahme	Bei Entlassung
*Hämatologische Diagnostik*				
Hämoglobin	11,5–15,0	g/dl	5,3	9,4
Hämatokrit	35–46	%	14,9	29,0
Erythrozyten	3,9–5,1	10^6^/µl	1,74	3,11
MCV	81–95	fl	86	93
MCH	26,0–32,0	pg	30,6	30,3
MCHC	32,0–36,0	g/dl	35,6	32,5
Thrombozyten	150–350	10^3^/µl	249	105
Leukozyten	4,0–11,0	10^3^/µl	7,40	2,38
Retikulozyten	≤25	‰	52	142
Retikulozytenproduktionsindex (RPI)	–	–	0,7	3,9
„Red cell distribution width“ (RDW)	11–15	%	–	18,2
*Differenzialblutbild*				
Metamyelozyten	–	%	1	–
Stabkernige	≤8	%	7	–
Segmentkernige	40–76	%	77	–
Lymphozyten	20–45	%	10	–
Monozyten	3–13	%	2	–
Basophile	≤2	%	2	–
*Klinische Chemie*				
Natrium	136–145	mmol/l	133	135
Kalium	3,5–4,6	mmol/l	4,3	3,7
Kreatinin	0,50–1,00	mg/dl	0,91	0,90
Harnsäure	2,6–6,0	mg/dl	7,4	–
Bilirubin, gesamt	0,3–1,2	mg/dl	1,7	0,7
Haptoglobin	0,14–2,58	g/l	0,09	0,69
Hämoglobin, freies	≤10	mg/dl	12	<5
Vitamin B12	187–883	ng/l	1137	–
Folsäure	3,1–20,5	µg/l	>40,0	–
Laktat-Dehydrogenase (LDH)	125–250	U/l	435	251

**Abb. 1:**
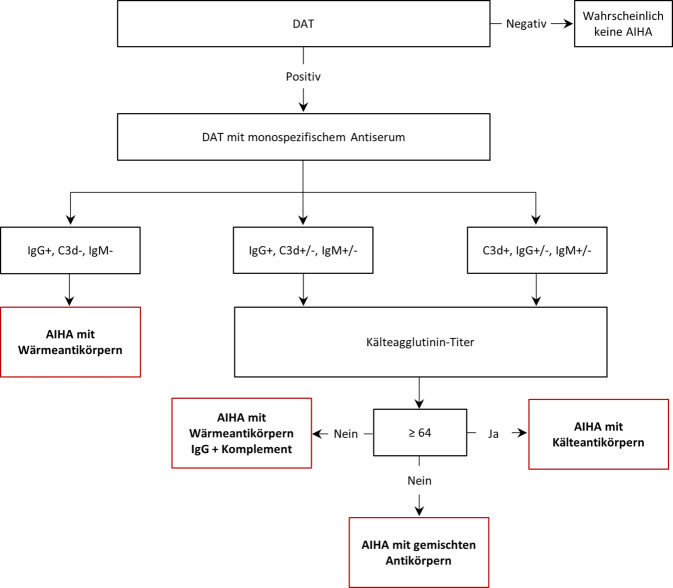
Diagnosealgorithmus beim Vorliegen einer Anämie mit Hämolyseparametern nach Durchführung eines direkten Antiglobulintests (DAT; Coombs-Test). Abbildung übersetzt und adaptiert aus [1]

Passend zur vorbekannten AIHA zeigte sich der direkte Antiglobulintest (DAT) positiv und ergab sowohl den Nachweis von Komplementfaktor C3d auf den Erythrozyten als auch von IgG (Abb. 1). Somit war hier von der Kombination einer intravasalen, komplementvermittelten Hämolyse (C3d) und einer extravasalen Hämolyse (IgG) auszugehen.

**Abb. 2:**
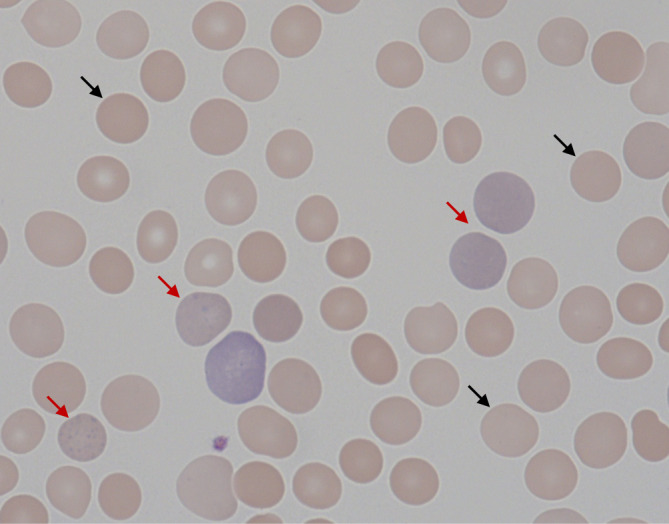
Blutausstrich mit Sphärozytose und Retikulozytose. Sphärozyten (*schwarze Pfeile*) imponieren als kugelförmige Erythrozyten ohne zentrale Aufhellung, eine mögliche Folge der Schädigung der Erythrozytenmembran durch Antikörper im Rahmen einer AIHA. Retikulozyten (*rote Pfeile*) sind als kernlose, polychromatische Zellen zu erkennen und Ausdruck einer reaktiv gesteigerten Erythropoese

Im Blutausstrich fielen sowohl eine Sphärozytose als auch eine Retikulozytose auf (Abb. 2). Initial war eine Erhöhung der Retikulozytenzahl auf 52 ‰ (NW ≤ 25 ‰) zu erheben. Der Retikulozytenproduktionsindex (RPI) lag jedoch nur bei 0,7 und brachte somit eine hyporegenerative Erythropoese zum Ausdruck. Beim Vorliegen einer AIHA ist prinzipiell jedoch infolge einer reaktiv gesteigerten Erythrozytenproduktion eher mit dem Vorliegen eines erhöhten RPI zu rechnen, da im physiologischen Gleichgewicht ein gesteigerter Abbau konsekutiv zur Steigerung der Erythropoese führt. Insofern ergaben die vorliegenden Befunde das Bild einer Dysbalance mit der ungewöhnlichen Kombination eines gesteigerten Abbaus bei gleichzeitig unzureichender Produktion von Erythrozyten. Weiterhin ergab sich mikroskopisch kein Verdacht einer mikroangiopathischen hämolytischen Anämie (MAHA), die beim Vorliegen von Hämolyseparametern in Kombination mit einer Anämie und Thrombozytopenie differenzialdiagnostisch in Betracht gezogen werden muss.

Einer verminderten hämatopoetischen Kapazität können vielfältige Ursachen zugrunde liegen. Dazu zählen Knochenmarkinfiltrationen im Rahmen maligner Erkrankungen oder Knochenmarkaplasien unterschiedlicher Ätiologie. Von einer erneuten Knochenmarkpunktion wurde bei fehlendem Hinweis auf eine hämatologische Grunderkrankung Abstand genommen. Zur differenzialdiagnostischen Abklärung einer möglichen Aplasie erfolgt u. a. eine erweiterte infektionsserologische Diagnostik. Hier zeigte sich ein positiver Befund für IgM- und IgG-Antikörper gegen Parvovirus B19. Eine ergänzende PCR-Analyse auf Parvovirus-B19-DNA ergab einen Nachweis von 6,0 · 10^7^ Kopien/ml und somit die Diagnose einer aktiven Infektion mit Parvovirus B19. Eine akute Infektion kann zu einer Insuffizienz der Erythropoese mit dem Bild einer „pure red cell aplasia“ (PRCA) führen. Somit war der vorliegende Befund einer hyporegenerativen Erythropoese im Rahmen der AIHA durch das gleichzeitige Vorliegen einer Parvovirus-B19-Infektion gut erklärlich.

## Diagnose

Autoimmunhämolytische Anämie vom Wärmeantikörpertyp mit hyporegenerativer Erythropoese bei insuffizienter Hämatopoese im Rahmen einer Parvovirus-B19-Infektion

## Therapie und Verlauf

**Abb. 3:**
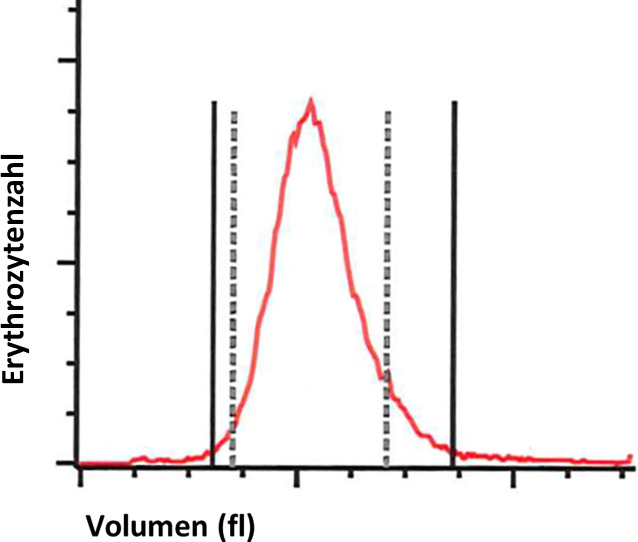
Erhöhte Erythrozytenverteilungsbreite (red cell distribution width [RDW]) im Rahmen der hyperregenerativen Erythropoese. Abbildung: UMG-Labor

**Abb. 4:**
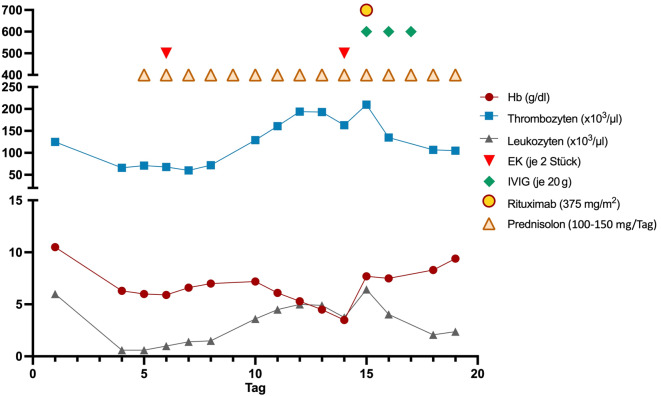
Blutbild (Hb, Leukozyten, Thrombozyten) und therapeutische Maßnahmen im Verlauf des stationären Aufenthalts

Auf die initial extern eingeleitete Therapie einer intravenösen Gabe von Glukokortikoiden zeigte sich auch nach Dosiserhöhung keinerlei Ansprechen. Bei rasch fortschreitendem Hb-Abfall ergab sich die Notwendigkeit der Transfusion von Erythrozytenkonzentraten als Notfallmaßnahme. Hierauf war eine gute Stabilisierung des Allgemeinzustands zu beobachten. Aufgrund der steroidrefraktären AIHA erfolgte anschließend die Gabe von Immunglobulinen über insgesamt drei Tage sowie eine Applikation des Anti-CD20-Antikörpers Rituximab. Hierunter zeigte sich der Hb-Wert zunächst stabil und schließlich steigend. Parallel waren die Hämolyseparameter deutlich rückläufig. Die Retikulozytenzahl stieg zuletzt auf 142 ‰ mit einem RPI von 3,9 im Sinne einer zunehmenden Regeneration der Erythropoese. Dazu passend war nun auch der RDW-Wert („red cell distribution width“; Erythrozytenverteilungsbreite) auf 18 % erhöht (normal: 11–15 %; Abb. 3). Während die Leukozyten zuvor unter der Steroidtherapie angestiegen waren, kam es nun a.e. als Reaktion auf die Gabe von Rituximab zu einem erneuten Abfall. Die Patientin berichtete von einer guten Beschwerdebesserung (Abb. 4).

## Diskussion

**Abb. 5:**
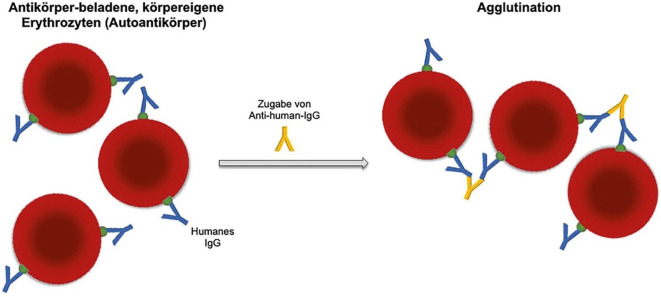
Direkter Antiglobulintest (DAT; direkter Coombs-Test) zum Nachweis von Antikörpern gegen Erythrozyten. IgG-Autoantikörper binden an erythrozytäre Antigene. Die im Coombs-Serum enthaltenen Antikörper gegen humane IgG-Antikörper binden an diese Autoantikörper und führen zur Agglutination. Abbildung übernommen aus [9]

Bei der autoimmunhämolytischen Anämie vom Wärmetyp kommt es bei Körpertemperatur zur Bindung von IgG-Wärmeautoantikörpern an erythrozytären Antigenen. Diese opsonierten Erythrozyten werden nachfolgend hauptsächlich in der Milz durch das mononukleäre Phagozytensystem abgebaut, teilweise nach Umwandlung zu Sphärozyten ([1]; Abb. 2). Laborchemisch zeigen sich die klassischen Hämolyseparameter sowie eine Retikulozytose als Ausdruck einer reaktiv gesteigerten Erythropoese. Diagnostisch wegweisend ist der Nachweis der an Erythrozyten gebundenen Antikörper und/oder Komplementfaktoren im direkten Coombs-Test (Abb. 5). Im Fallbeispiel gab die initiale hyporegenerative Erythropoese sowie Entwicklung der ausgeprägten Panzytopenie Anlass zur weiteren Umfelddiagnostik. Diese ergab schließlich den Nachweis einer aktiven Parvovirus-B19-Infektion.

Das Parvovirus B19 ist ein unbehülltes DNA-Virus, das per Tröpfcheninfektion übertragen wird. Während das Virus im Kindesalter die typischen Ringelröteln (Erythema infectiosum) verursacht, führt es bei Erwachsenen häufig zu Arthropathien [2]. Da das Parvovirus B19 bevorzugt erythroblastäre Zellen im Knochenmark befällt, kommt es durch den Untergang dieser Vorläuferzellen zu einer passageren Anämie. Die Zerstörung ist nicht antikörpervermittelt, sodass der Coombs-Test negativ ausfällt. Das Parvovirus B19 wirkt sich insbesondere auf die Erythropoese aus, kann aber auch die weiteren Zellreihen hin zu einer passageren Myelosuppression beeinflussen [3]. In seltenen Fällen kann dies auch zu aplastischen Krisen führen [4]. Weiterhin wird das Parvovirus B19 in Fallberichten wiederholt als möglicher Trigger einer AIHA beschrieben, wenngleich die Ansätze zur möglichen Pathogenese hypothetisch bleiben [5, 6].

Im vorliegenden Fall kann die Parvovirus-B19-Infektion sowohl als direkter wie indirekter Auslöser der schweren Anämie diskutiert werden. Einerseits kommt das Virus als möglicher Trigger der AIHA in Betracht, andererseits bedingt die Virusinfektion durch die Störung der Erythropoese selbst direkt eine zunehmende Anämie. Ähnliche Fallkonstellationen mit dem Vorliegen einer Parvovirus-B19-Infektion bei komplikativen Verläufen einer AIHA wurden insbesondere bei Kindern beschrieben [7, 8].

## Fazit für die Praxis

- Die Koinzidenz einer AIHA mit einer Parvovirus-B19-Infektion kann zu einem atypischen klinischen Bild führen und die Diagnosestellung erschweren, insbesondere bei gleichzeitig auftretender Panzytopenie.
- Eine hyporegenerative Erythropoese bei AIHA ist untypisch und sollte Anlass zur erweiterten Differenzialdiagnostik geben, z. B. hinsichtlich infektiöser oder medikamentöser Ursachen einer Knochenmarksuppression.
- Eine Panzytopenie im Rahmen einer Parvovirus-B19-Infektion ist möglich und resultiert aus einer passageren Myelosuppression.
- Eine serologische und molekulare Diagnostik auf Parvovirus B19 (IgM, IgG, PCR) sollte bei ungeklärter Anämie mit Knochenmarkinsuffizienz frühzeitig erfolgen.
- Bei Parvovirus-B19-Infektion ist der direkte Coombs-Test in der Regel negativ, da die Anämie nicht antikörpervermittelt ist. Ein positiver Coombs-Test spricht für eine AIHA. Bei gleichzeitigem Virusnachweis muss an eine Koinzidenz gedacht werden.
